# Host associations and genomic diversity of *Borrelia hermsii* in an endemic focus of tick-borne relapsing fever in western North America

**DOI:** 10.1186/s13071-016-1863-0

**Published:** 2016-11-10

**Authors:** Tammi L. Johnson, Robert J. Fischer, Sandra J. Raffel, Tom G. Schwan

**Affiliations:** 1 Laboratory of Zoonotic Pathogens, Rocky Mountain Laboratories, National Institute of Allergy and Infectious Diseases, National Institutes of Health, Hamilton, MT USA; 2 Division of Vector-Borne Diseases, Centers for Disease Control and Prevention, Fort Collins, CO USA; 3 Laboratory of Virology, Rocky Mountain Laboratories, National Institute of Allergy and Infectious Diseases, National Institutes of Health, Hamilton, MT USA

**Keywords:** *Ornithodoros hermsi*, Tick-borne zoonosis, Montana, Argasidae

## Abstract

**Background:**

An unrecognized focus of tick-borne relapsing fever caused by *Borrelia hermsii* was identified in 2002 when five people became infected on Wild Horse Island in Flathead Lake, Montana. The terrestrial small mammal community on the island is composed primarily of pine squirrels (*Tamiasciurus hudsonicus*) and deer mice (*Peromyscus maniculatus*), neither of which was known as a natural host for the spirochete. Thus a 3-year study was performed to identify small mammals as hosts for *B. hermsii.*

**Methods:**

Small mammals were captured alive on two island and three mainland sites, blood samples were collected and examined for spirochetes, and serological tests performed to detect anti-*B. hermsii* antibodies. *Ornithodoros hermsi* ticks were collected and fed on laboratory mice to assess infection. Genomic DNA samples from spirochetes isolated from infected mammals and ticks were analyzed by multilocus sequence typing.

**Results:**

Eighteen pine squirrels and one deer mouse had detectable spirochetemias when captured, from which 12 isolates of *B. hermsii* were established. Most pine squirrels were seropositive, and the five species of sciurids combined had a significantly higher prevalence of seropositive animals than did the other six small mammal species captured. The greater diversity of small mammals on the mainland in contrast to the islands demonstrated that other species in addition to pine squirrels were also involved in the maintenance of *B. hermsii* at Flathead Lake. *Ornithodoros hermsi* ticks produced an additional 12 isolates of *B. hermsii* and multilocus sequence typing identified both genomic groups of *B. hermsii* described previously, and identified a new genomic subdivision. Experimental infections of deer mice with two strains of *B. hermsii* demonstrated that these animals were susceptible to infection with spirochetes belonging to Genomic Group II but not Genomic Group I.

**Conclusions:**

Pine squirrels are the primary hosts for the maintenance of *B. hermsii* on the islands in Flathead Lake, however serological evidence showed that numerous additional species are also involved on the mainland. Future studies testing the susceptibility of several small mammal species to infection with different genetic types of *B. hermsii* will help define their role as hosts in this and other endemic foci.

## Background

Tick-borne relapsing fever is a zoonotic disease endemic in regions of Africa, central Asia and the Americas [1–3]. The illness is caused by spirochetes transmitted by the bite of infected *Ornithodoros* species of ticks or through coxal fluid secreted by the tick while still on their host [2, 4]. In North America, relapsing fever associated with argasid ticks is caused by three species of spirochetes, *Borrelia hermsii*, *Borrelia turicatae* and *Borrelia parkeri.* Each species of spirochete is transmitted by their respective tick vector, *Ornithodoros hermsi*, *Ornithodoros turicata* and *Ornithodoros parkeri* [1, 2]. The basis of this strict host specificity between spirochete and tick is not known.

*Borrelia hermsii* is the primary cause of human tick-borne relapsing fever in North America [5]. The spirochete is maintained in enzootic cycles involving the tick vector *O. hermsi* and small mammal hosts. The tick and spirochete have a wide geographical distribution in the western part of the continent, ranging from Southern California to Southern British Columbia, and east to the Rocky Mountains in Colorado [5–10]. However, endemic foci of infection are primarily restricted to coniferous forests at elevations ranging from just under 900 m to 2,000 m and higher. People usually become infected at night while sleeping in rustic cabins that are infested with the nocturnal, fast feeding ticks.

Despite the wide geographical distribution of these ticks and spirochetes, the numbers of reported human cases are relatively few, due in part to infected patients not being diagnosed correctly [11]. From 1990 to 2011, 96 % of the 504 confirmed and suspected cases of relapsing fever reported to CDC originated in areas endemic for *B. hermsii*; the remaining 4 % were from Texas where *B. turicatae* is found [12].

During the summer of 2002, five of 20 people contracted relapsing fever while attending a family reunion at a cabin on Wild Horse Island in Flathead Lake, Lake County, Montana [13, 14]. *Borrelia hermsii* was isolated from two of the patients and *O. hermsi* ticks were collected from a rodent nest removed from the cabin’s attic. These findings were the first isolation of *B. hermsii* and the first collection of *O. hermsi* in Montana, which identified a previously unknown focus of tick-borne relapsing fever in the inland northwest of the United States. Two years later, three more people were infected with *B. hermsii* while staying in another cabin on the island, not far from where the initial outbreak occurred. The five isolates of *B. hermsii* that originated from the patients infected on the island were characterized by multi-locus sequence typing (MLST) that targeted the *16S rRNA*, *flaB*, *gyrB* and *glpQ* genes and 16S rDNA-23S rDNA intergenic spacer (IGS) [8]. Previous work in our laboratory using these genetic targets defined two genomic groups (GGI and GGII) of *B. hermsii* from numerous locations in western North America [15]. Among the *B. hermsii* that infected the Wild Horse Island patients, one isolate belonged to GGI while the other four isolates belonged to GGII [8]. Of note, the three patients infected in 2004 had slept in the same bed, and from one patient we isolated a GGI spirochete while from the other two patients we isolated GGII spirochetes [8]. This finding was our first observation that members of both genomic groups of *B. hermsii* were present in the same endemic focus, and could coexist in one or more ticks infesting the same cabin.

Given the recent number of people that contracted relapsing fever on the island, and the lack of knowledge regarding the animals involved in maintaining this focus of infection, we initiated a 3-year study to examine the prevalence, distribution and genetic diversity of *B. hermsii* on Wild Horse Island and other locations in the Flathead Lake area. We show that pine squirrels (*Tamiascuirus hudsonicus*) and other members of the squirrel family (Sciuridae) are predominant hosts for the spirochetes, the tick vector is established at multiple sites, and that genetically diverse *B. hermsii* are sympatric in this endemic focus.

## Methods

### Study area

**Fig. 1:**
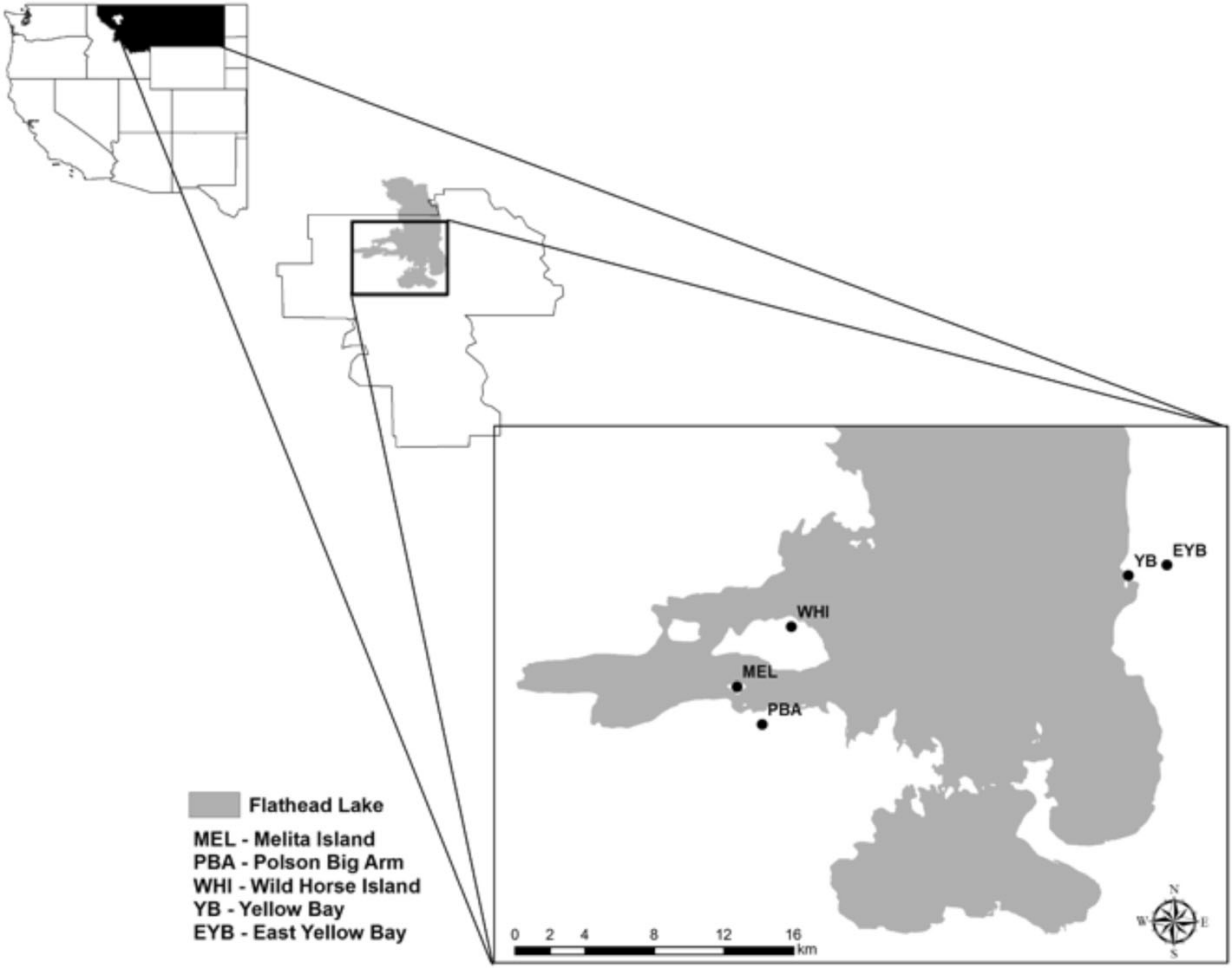
Map of Flathead Lake, Lake County, Montana (inset) with locations of study sites

Flathead Lake is in the southern region of Flathead Valley immediately west of the Mission Mountains in Lake County, Montana (47°54′24''N, 114°06′57''W) and is the largest natural freshwater lake in the western United States with a surface area of approximately 496 km^2^ (Fig. 1). The elevation of the lake above sea level varies seasonally between 878 to 882 m. Wild Horse Island is the largest island in the lake (~875 ha) and is a day-use only Montana State Park except for 56 privately owned lots of 0.2 or 0.4 ha located on the perimeter of the island. There are no permanent residents but occasionally individuals overwinter there. Melita Island lies between Wild Horse Island and the southwestern shore of the lake and is owned by the Boy Scouts of America. This small island (26 ha) is the site for scouting activities during the summer. Flathead Lake Biological Station at Yellow Bay is a permanent research and teaching facility on the eastern shore of the lake and is owned by the University of Montana. East Yellow Bay is a privately owned, undeveloped plot of land above and to the east of Yellow Bay. Polson/Big Arm area is also on the mainland near the southwest shore of the lake in the Flathead Indian Reservation. The habitat targeted for mammal trapping at each site was mixed coniferous forest composed primarily of yellow pine (*Pinus ponderosa*) and Douglas fir (*Pseudotsuga menziesii*) on the islands, and also western larch (*Larix occidentalis*), lodgepole pine (*Pinus contorta*), western hemlock (*Tsuga heterophylla*) and black cottonwood (*Populus balsamifera*) on the mainland. Historically, the coldest and warmest months are January and July, respectively. The mean low and high temperatures for Kalispell, MT, in January are -9.8 and -1.6 °C and in July, 10.1 and 27.7 °C (Western Regional Climate Center, Reno, NV, USA).

### Small mammal trapping

Small mammals were trapped from May to August during 2008 to 2010. Animals were captured with large Sherman live traps (7.6 × 8.9 × 22.9 cm) (HB Sherman Traps, Tallahassee, FL, USA) and Tomahawk wire mesh live traps (15.2 × 15.2 × 61 cm) (Tomahawk Live Trap Company, Tomahawk, WI, USA). Pine squirrels were initially targeted because of their presumptive but not proven role as a primary host for *B. hermsii*. Traps were initially set in transects with each location comprising one Tomahawk and two Sherman traps. Bait varied with a mixture of rolled oats, peanuts and pine nuts. During the final year, a 12 × 12 grid with 15 m spacing was established at each site and one Sherman trap was placed at each grid stake. Each site was trapped for four consecutive days and traps were checked every 4 h during daylight. We trapped 4,476 trap nights (TN) on Wild Horse Island, 2,632 TN at Yellow Bay, 2,032 TN at East Yellow Bay, 2,032 TN on Melita Island, and 1,732 TN at Polson/Big Arm.

Animals were captured alive and anesthetized in an inhalation chamber with Isoflurane (Fluriso) (Vet ONE, MWI Veterinary Supply, Boise ID, USA). Individuals were identified to species (except *Microtus* sp. and *Sorex* sp.) and gender, weighed to the nearest 0.1 g, and standard body measurements taken. Blood samples were collected from the tail or saphenous vein of the squirrels and chipmunks (up to 600 μl) and from the retro-orbital sinus of the smaller species (up to 150 μl). Venous samples from the sciruids were mixed with 3 μl heparin (Heparin sodium, 1,000 USP Units/ml; Baxter Healthcare Corp., Deerfield, IL, USA). Retro-orbital sinus samples were collected with Drummond Hemato-Clad Heparinized Mylar-Wrapped Hematocrit tubes (Fisherbrand, Fisher Scientific, Pittsburgh, PA, USA). Each sample was put in a 2 ml screw-cap plastic vial and chilled until testing.

Each animal was implanted subcutaneously with a 9 mm passive integrated transponder (PIT) tag (Biomark, Inc., Boise, ID, USA) for identification when recaptured. Animals were allowed to recover in the trap before being released at the point of capture. Animals recaptured during the same week were identified and recorded but blood was not sampled again.

### Sample and laboratory analyses

Thin smears of blood were prepared in the field with 2.5 μl of whole blood. In the laboratory the smears were fixed with 100 % methanol and stained with a modified Wright-Giemsa stain following the manufacturer’s instructions (Quick III Statpak differential stain kit, Astral Diagnostics, Inc., West Deptford, NJ, USA). Slides were examined with a light microscope (Nikon Eclipse E800, Nikon Instruments, Melville, NY, USA) at 600× magnification using a 60× oil immersion objective lens. Fifty fields were examined on each slide for the presence of spirochetes. Wet drops were prepared by placing 3 μl of blood on the slide, covered with a 22 × 22 mm coverslip, and examining 50 fields with a dark-field microscope (Nikon Eclipse E600) at 400× magnification. If live spirochetes were seen, up to 200 μl of rodent blood was inoculated into a laboratory mouse (RML Colony Strain, *Mus musculus*) via intraperitoneal injection. Laboratory mice were monitored daily for the presence of spirochete infection by examining approximately 3 μl of blood obtained from the tail vein by nicking the tip of the tail. When ten or more spirochetes were observed per microscope field, we euthanized the mouse, collected blood by intracardiac puncture, and placed 50 μl of infected blood into 5 ml of BSK-H medium (Sigma-Aldrich Corp., St. Louis, MO, USA) supplemented with 12 % rabbit serum and incubated at 33 °C.

All DNA sequences presented herein except for the infected deer mouse (*Peromyscus maniculatus*) were determined from genomic DNA samples purified from spirochetes isolated in culture using a phenol-chloroform extraction method described previously [16]. When isolates were established, DNA was also extracted from the whole-blood sample from the infected pine squirrel using the QIAGEN DNeasy Blood and Tissue Kit (Qiagen, Valencia, CA, USA) according to the manufacturer’s instructions. PCR was performed with purified DNA samples using Promega GoTaq Flexi DNA polymerase (Promega Corp, Madison, WI, USA) and primers for the *B. hermsii 16S rRNA*, *flaB, gyrB*, *glpQ* and *vtp* genes [8, 15]. The PCR conditions included an initial denaturation at 96 °C for 3 min, followed by 35 cycles of 94 °C for 30 s, 55 °C for 30 s and 72 °C for 2 min 30 s, followed by a final extension of 7 min at 72 °C. Samples were analyzed by electrophoresis in 1 % agarose gels and positive samples were submitted for Sanger sequencing for comparison to other laboratory isolates of *B. hermsii* in our collection.

Sequence chromatograms were visualized manually to confirm the identity of each base using Sequencher v. 5.0 (Gene Codes Corporation, Ann Arbor, MI, USA). Sequences were aligned using the CLUSTAL W function, trimmed and concatenated using the MegAlign program (Lasergene, DNASTAR, Inc., Madison, WI, USA). The phylogram of the four concatenated genes (*16S rRNA, flaB, gyrB, glpQ*; 5,188–5,203 bp) was created using MegAlign that utilizes the Kimura distance method, which results in a pair-wise genetic distance matrix used to create a neighbor-joining tree [17].

### Serology

Western blot analysis was performed to detect antibodies to relapsing fever spirochetes using a whole-cell lysate of *B. hermsii* DAH and a purified recombinant GlpQ protein from the same strain [18]. The protein preparations were separated by electrophoresis in Novex® 4–20 % Glycine Gels 1.0 mm (Invitrogen, Life Technologies, Grand Island, NY, USA), and transferred to nitrocellulose membranes using the iBlot® Gel Transfer Device according to the manufacturer’s instructions (Invitrogen, Life Technologies). Membranes were blocked in Tropix® I-BLOCK (Applied Biosystems, Life Technologies, Grand Island, NY, USA) at room temperature for 1 h. Serum samples were diluted 1:100 in 5 ml of I-BLOCK and incubated with the membrane at room temperature for 1 h. Membranes were removed from the serum samples, washed with I-BLOCK, and incubated with HRP-conjugated recombinant Protein A (1:4,000) (Invitrogen, Life Technologies). The membranes were washed in I-BLOCK for 2 h with four changes of the wash solution, and then developed for ~30 s using ECL® Western Blotting Detection Reagents (GE Healthcare, Little Chalfont, UK). Positive and negative control samples were obtained from infected and uninfected laboratory mice. A serum sample was considered positive if it contained antibodies that bound to eight or more proteins in the *B. hermsii* whole-cell lysate and to the purified GlpQ.

### Tick collection

We collected material from nest cavities in trees and snags, pine squirrel middens and ground burrows from Wild Horse Island, Melita Island, Yellow Bay, and East Yellow Bay. Material was sealed in plastic bags and returned to Rocky Mountain Laboratories for processing in Berlese extraction funnels. The *O. hermsi* ticks collected from this material were identified to stage and sex, and fed individually on laboratory mice to determine if they were infected with *B. hermsii.* Laboratory mice were monitored for infection and spirochetes were isolated from their blood as described above.

### Experimental infections of deer mice with *B. hermsii*

We used adult deer mice (*P. maniculatus rufinus*) from a RML colony that was founded with animals from a colony in New Mexico [19]. We experimentally infected these mice with *B. hermsii* belonging to GGI or GGII to determine if there was a difference in susceptibility of these animals to these genetically distinct spirochetes. First, we inoculated one deer mouse each intraperitoneally with 200 μl of PBS containing 2 × 10^5^ spirochetes of either *B. hermsii* DAH (GGI) or *B. hermsii* MTW (GGII) grown in BSK-H medium. Mice were monitored daily for spirochetemia for 21 days post-inoculation by nicking the tip of the tail and expressing ~5 μl of blood onto a glass microscope slide, spreading the sample under a 22 × 22 mm glass coverslip, and counting the spirochetes observed in 50 fields using a dark-field microscope at 400× magnification.

We next fed groups of infected *O. hermsi* nymphs on adult deer mice following the protocol described elsewhere [20]. Five deer mice were fed upon by 48, 51, 79, 87 or 88 ticks infected with *B. hermsii* DAH (GGI) and four deer mice were fed upon by 4, 7, 14 or 18 ticks infected with *B. hermsii* MTW (GGII) (a fifth animal died shortly after tick feeding). The density of spirochetes in the blood of these animals was determined for 21 days as described previously [21]. Briefly, 2.5 μl of blood from the tail vein was spread evenly within an etched circle (area = 71.22 mm^2^) on a glass microscope slide, dried at room temperature, stained with a Giemsa stain, and examined under a bright-field microscope at 600× magnification with a 60× oil immersion objective (area per field = 0.126 mm^2^). The number of spirochetes in 50 fields was counted and the total number of spirochetes per milliliter of deer mouse blood was estimated. Two white mice (*Mus musculus*, RML Laboratory Strain) were also fed upon by ticks infected with the two strains of spirochetes to ensure tick infection, as white mice are highly susceptible to infection with these two strains of *B. hermsii.* [9, 22].

### Statistics

The diversity of small mammal species at each site was defined by the Shannon-Wiener diversity index (H) (Eq. 1) and variance of H (Eq. 2):

1 $$ H = \varSigma {p}_i\left( \ln\ {p}_i\right) $$

2 $$ var(H) \approx \frac{{\displaystyle {\sum}_{i=1}^s}{p}_i{\left( \ln {p}_i\right)}^2 - {\displaystyle {\sum}_{i=1}^s}{\left({p}_i\  \ln {p}_i\right)}^2}{N} - \frac{S-1}{2{N}^2} $$

where *p*_*i*_ is the proportion of individuals of species *i*, *S* is the total number of species, and *N* is the total number of individuals [23, 24]. The Shannon-Wiener diversity index accounts for both species richness and evenness of captures. During this study, trapping efforts varied among sites and years, so we were unable to test for year effects and weighted all statistical analyses accordingly. To determine if some species of rodents had significantly more seropositive individuals than other species to relapsing fever spirochetes, we used an analysis of variance (ANOVA). The analysis was weighted by the number of captures for each species and included the site as a fixed effects variable; the assumption of normality was confirmed using the Shapiro-Wilk test. We also used weighted ANOVA [weighted by var (H)] to test for significant differences in species diversity among study sites. We used weighted [var (H)] linear regression to test for a significant relationship between species diversity and seroprevalence over sites and years. The Tukey’s method was used for all multiple comparison testing. Statistical analyses were conducted with the statistical package R v 2.11 and GraphPad Prism v 5; an alpha level of 0.05 was used to test for statistical significance.

## Results

### Small mammals captured

In total, we captured 666 small mammals during 12,904 trap nights at five study sites from 2008 to 2010 (Tables 1 and 2) and included 11 species. Deer mice (*P. maniculatus*) were the most frequently captured animals at all sites where they occurred, and these mice comprised over half (54.8 %) of the animals trapped. Pine squirrels (*T. hudsonicus*) were second in number captured and comprised 24.3 % of the animals we trapped. The remaining nine species together comprised 21 % of the captures but separately each species represented only a small percentage of the animals sampled.

**Table 1:** Species and numbers of small mammals captured and seropositive for *B. hermsii* antibodies on island sites

	Wild Horse Island			Melita Island		Total	
Species	2008	2009	2010	2009	2010	No. positive/No. tested	% Positive
*P. maniculatus*	3/52^a^	2/10	0/52	NP^b^	NP	5/114	4.4
*T. hudsonicus*	24/26	12/12	3/3	7/7	19/25	65/73	89.0
*Microtus* sp.	0	0/1	0	0/1	0/2	0/2	0
Total	27/78	14/23	3/55	7/8	19/25	70/189	37.0

^a^Number positive/number tested

^b^NP, not present

**Table 2:** Species and numbers of small mammals captured and seropositive for *B. hermsii* antibodies at the mainland sites

	Yellow Bay			East Yellow Bay		Polson-Big Arm		Totals	
Species	2008	2009	2010	2009	2010	2008	2010	No. positive/No. tested	% Positive
*P. maniculatus*	4/67^a^	2/33	2/64	3/17	2/27	0/7	2/36	15/251	6.0
*T. hudsonicus*	12/16^b^	20/32^c^	18/28^d^	5/5	0	0	0	55/81	67.9
*Microtus* sp.	2/3	0/4	1/24	0/1	0/5	0	0/12	3/49	6.1
*T. ruficaudus*	2/3	0/1	0	10/15	3/6	1/1	5/18	21/44	47.7
*T. amoenus*	2/2	0	0	0	0	0/2	0/1	2/5	40.0
*G. sabrinus*	1/2	6/6	1/1	2/2	0	0	0	10/11	90.9
*Z. princeps*	1/5	0/3	0/3	0	0/1	0	0	1/12	8.3
*Sorex* sp.	1/1	0/4	1/1	0	0	0	0/1	2/7	28.6
*S. columbianus*	0	0	0	0	0	0	2/4	2/4	50.0
*M. erminea*	0	0/1	0/2	0	0	0	0	0/3	0
*N. cinerea*	0	0/1	0	0	0/1	0	0	0/2	0
Totals	25/99	28/85	23/123	20/40	5/40	1/10	9/72	111/469	23.7

^a^Number positive/number tested

^b^15 individuals with 1 recapture

^c^25 new individuals and 7 recaptures from previous year

^d^27 individuals with 1 animal from previous year recaptured twice

**Fig. 2:**
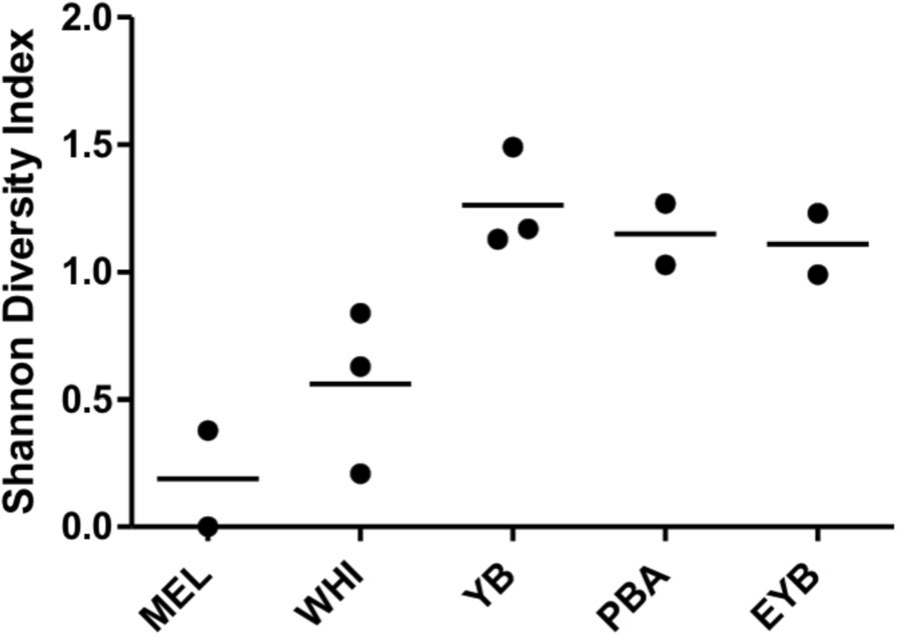
Species diversity of small mammals among the five field sites sampled at Flathead Lake, Montana. Each dot represents the Shannon-Wiener diversity index for the site for each of the 2 or 3 years sampled. Species diversity on the two islands was significantly less than at the three mainland sites (ANOVA: *F*_(4,7)_ = 8.248, *P* = 0.009; horizontal bars represent the mean indices). *Abbreviations*: MEL, Melita Island; WHI, Wild Horse Island; YB, Yellow Bay; PBA, Polson - Big Arm; EYB, East Yellow Bay

Species diversity of the mammals differed significantly among study sites (ANOVA: *F*_(4,7)_ = 8.248, *P* = 0.009) with the two islands having far fewer species than the mainland (Fig. 2; Tables 1 and 2). Melita Island was inhabited almost exclusively by pine squirrels. No deer mice were captured and prior to our sampling, no voles (*Microtus* sp.) were known on this island; we captured one individual. Both Melita and Wild Horse Islands lacked most of the species we captured on the mainland, which included two species of chipmunks (*Tamias ruficaudus* and *Tamias amoenus*), northern flying squirrels (*Glaucomys sabrinus*), Columbian ground squirrels (*Spermophilus columbianus*), bushy-tailed woodrats (*Neotoma cinerea*), jumping mice (*Zapus princeps*), shrews (*Sorex* sp.) and short-tailed weasels (*Mustela erminea*).

### *Borrelia hermsii* infection in mammals

Active *B. hermsii* infections were detected microscopically in 18 pine squirrels (9 male, 9 female) captured on Wild Horse Island (*n* = 4), Melita Island (*n* = 7), and at Yellow Bay (*n* = 7), and in a single deer mouse captured on Wild Horse Island. Thus, 2.9 % (19 of 666) of the animals had detectable infections at the time of their capture.

Blood samples from the infected pine squirrels were inoculated into laboratory mice, which produced sufficiently high cell densities of spirochetes in the blood of 12 of the mice that resulted in their continuous growth and isolation in liquid medium. However, spirochetes detected in 6 other pine squirrels produced low levels of infection in mice and we were unable to isolate them in culture. We were not allowed to inoculate the infected deer mouse blood into laboratory mice in the RML animal facility due the concern of possible Sin Nombre virus infection, thus we performed PCR analysis on a heat-treated sample (see below).

### Serological tests for anti-*B. hermsii* antibodies

**Fig. 3:**
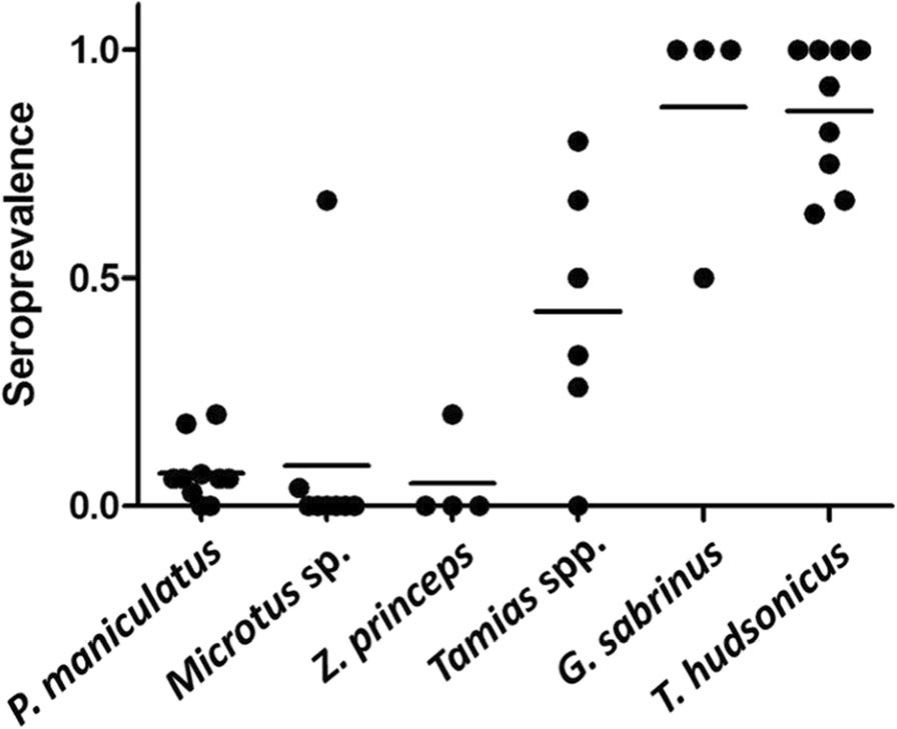
Seroprevalence of anti-*Borrelia hermsii* antibodies in serum samples collected from small mammals at Flathead Lake, Montana. Each dot represents the percentage of individuals seropositive out of the total number sampled at one site for each of the 2 or 3 years sampled. The seroprevalence for *Tamiasciurus hudsonicus*, *Glaucomys sabrinus*, and the *Tamias* species combined was significantly higher than for the other species (ANOVA, *F*_(5,35)_ = 29.080, *P* < 0.0001; horizontal bars represent the mean values). Species with less than ten individuals captured per location and season were omitted. Seroprevalence of 1.0 = 100 %

Immunoblot analysis of 658 serum samples collected from all species and locations showed that 27.5 % of the animals were seropositive (Tables 1 and 2), indicating previous infections with *B. hermsii*. All sites had positive animals although the prevalence of antibody-containing individuals varied among the locations with Polson/Big Arm having the lowest (12.2 %) and Melita Island the highest (78.8 %) proportion of positive animals. Cumulatively, the sciurids, which included the pine squirrels, yellow-pine and red-tailed chipmunks, northern flying squirrels, and Columbian ground squirrels, had 12 times more seropositive individuals (155 of 218 positive = 71.1 %) than did the other species combined (26 of 440 positive = 5.9 %) (ANOVA, *F*_(5,35)_ = 29.080, *P* < 0.0001). Pine squirrels, the second most frequently captured species, and flying squirrels, dominated the other species with their high seroprevalences of 77.9 and 90.9 %, respectively (Fig. 3). Deer mice had the lowest percentage of seropositive individuals (5.5 %), excluding the very few seronegative short-tailed weasels and bushy-tailed woodrats we also captured. Despite our finding spirochetes in only pine squirrels and one deer mouse, the serological results demonstrate that many other species of small mammals were involved to a lesser degree as hosts for both the ticks and spirochetes in the areas we sampled.

### Spirochete exposure and diversity of host species

**Fig. 4:**
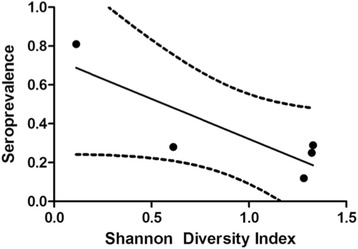
Seroprevalence and species diversity among the small mammals captured at Flathead Lake, Montana. Each dot represents the percentage of individuals for all species seropositive out of the total number captured at each of the five sites for the 2 or 3 years sampled. The greater speroprevalence in relation to lower Shannon-Wiener diversity index was marginally insignificant (*R* ^2^ = 0.69, *P =* 0.052) and influenced most by data for Melita Island (upper left dot). The dashed lines represent the 95 % confidence intervals

As presented above, the species diversity on the islands was significantly less than on the mainland (Fig. 2). However, pine squirrels, which were abundant on both islands, were also common at two mainland sites (Yellow Bay and East Yellow Bay), and in all locations these animals were clearly involved in the infectious cycle of *B. hermsii*. Therefore, past infections for pine squirrels on the islands versus the mainland were compared. Pine squirrels on the islands had a greater percentage of their population with antibodies compared to the pine squirrels on the mainland (Tables 1 and 2). Although this difference was not statistically significant, the larger proportion of pine squirrels exposed to spirochetes on the islands where other hosts were either rare or absent, is interesting. When all species and sites were compared, there was an inverse relationship between species diversity and seroprevalence at a site, as overall the seroprevalence was higher where the host species diversity was less (Fig. 4), although not statistically significant (*R* ^*2*^ = 0.69, *P* = 0.052, *n* = 5).

### Spirochete isolation from *O. hermsi* ticks

We collected 31 *O. hermsi* (24 nymphs, three males, four females) from six nests taken from Yellow Bay, Melita Island and Wild Horse Island. These ticks were fed individually on laboratory mice, which resulted in 13 of the mice becoming infected. Ticks that transmitted spirochetes to mice included nine nymphs, two males, and two females. Based on the tick feeding experiments, 42 % (13 of 31) of the ticks were infected, although this may underrepresent the prevalence of infection, as infected ticks may not always transmit spirochetes when feeding. From the 13 infected mice, we established 12 isolates of *B. hermsii* in culture that originated from ticks collected on Wild Horse Island, Melita Island, and at Yellow Bay.

### Genomic characterization of spirochetes

**Fig. 5:**
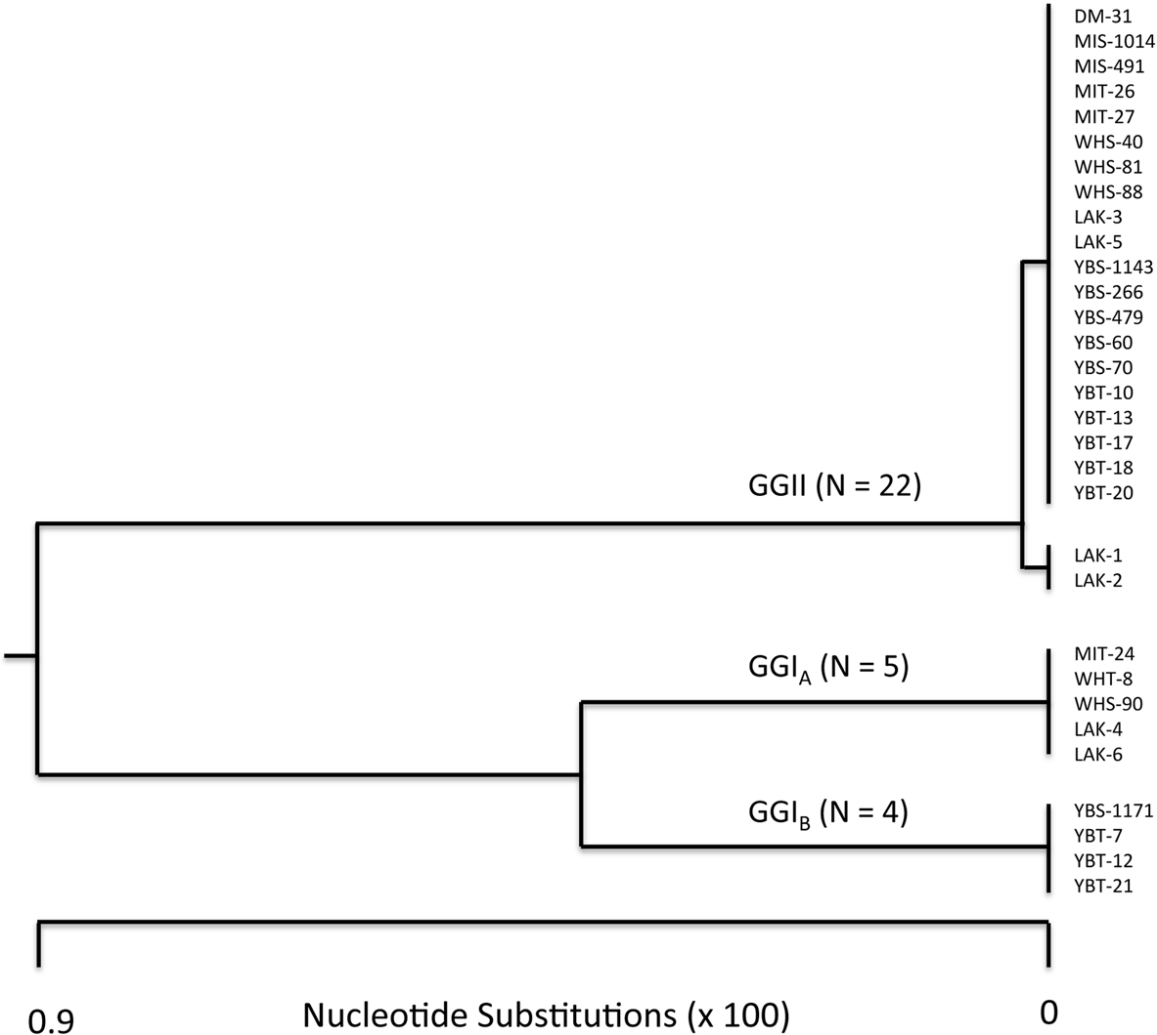
Phylogram for *Borrelia hermsii* originating from Flathead Lake, Montana. The tree was constructed with concatenated DNA sequences of four genes (*16S rRNA, flaB, gyrB* and *glpQ*; 5,188–5,203 bp) for spirochetes infecting pine squirrels, ticks, one deer mouse, and from human patients from previous studies. Sequences were aligned using CLUSTAL W and the tree was constructed using the neighbor-joining algorithm [17]

Genomic DNA samples from spirochetes established in culture during this study were examined by MLST and compared to isolates of spirochetes that originated from human relapsing fever patients infected previously on Wild Horse Island [8, 13]. In all, the analysis included 31 samples of *B. hermsii* that originated from 12 pine squirrels, 12 *O. hermsi* ticks, one deer mouse and six people (Table 3). Sequence alignments of the four concatenated genes (*16S rRNA, flaB, gyrB* and *glpQ*, totaling 5,188–5,203 bp) segregated the spirochetes into the two primary genomic groups described previously (GGI and GGII) (Fig. 5) [15]. Both analyses supported the more recent division of GGI spirochetes into two subdivisions, GGI_A_ and GGI_B_ [25]. All GGI_A_ spirochetes were identical to each other for the 5,197 bp examined. The GGI_B_spirochetes were also identical to each other for the 5,188 bp determined, which was slightly less than for the GGI_A_ spirochetes due to a 9-bp deletion in the *flaB* sequence. GGII spirochetes were nearly all identical for the 5,203 bp determined but two samples, LAK-1 and LAK-2, varied from all others at just two positions. DNA sequences have been deposited in GenBank with the following accession numbers: *16S rRNA* (KX171899–KX171924); *flaB* (KX171792–KX171817); *gyrB* (KX171873–KX171898); *glpQ* (KX171818–KX171843).

**Table 3:** Isolates of *Borrelia hermsii* established from Flathead Lake, Montana, by location, year of isolation, biological source, isolate name, genomic group, and Vtp type

Location	Year	Source	Name	Group	Vtp Type
YB	2008	Squirrel^a^	YBS 60	GGII	5
YB	2008	Squirrel	YBS 70	GGII	5
YB	2009	Squirrel	YBS 266	GGII	5
YB	2010	Squirrel	YBS 479	GGII	5
YB	2010	Squirrel	YBS 1143	GGII	5
YB	2010	Squirrel	YBS 1171	GGI_B_	7
YB	2010	Tick^b^	YBT 7	GGI_B_	7
YB	2010	Tick	YBT 10	GGII	5
YB	2010	Tick	YBT 12	GGI_B_	7
YB	2010	Tick	YBT 13	GGII	5
YB	2010	Tick	YBT 17	GGII	5
YB	2010	Tick	YBT 18	GGII	5
YB	2010	Tick	YBT 20	GGII	5
YB	2010	Tick	YBT 21	GGI_B_	7
WHI	2008	Squirrel	WHS 40	GGII	5
WHI	2008	Squirrel	WHS 81	GGII	5
WHI	2008	Squirrel	WHS 88	GGII	5
WHI	2008	Squirrel	WHS 90	GGI_A_	6
WHI	2008	Mouse^c^	DM 31	GGII	5
WHI	2010	Tick	WHT 8	GGI_A_	6
WHI	2002	Human	LAK-1	GGII	1
WHI	2002	Human	LAK-2	GGII	1
WHI	2004	Human	LAK-3	GGII	5
WHI	2004	Human	LAK-4	GGI_A_	6
WHI	2004	Human	LAK-5	GGII	5
WHI	2009	Human	LAK-6	GGI_A_	6
MEL	2010	Squirrel	MIS 1014	GGII	5
MEL	2010	Squirrel	MIS 491	GGII	5
MEL	2010	Tick	MIT 26	GGII	5
MEL	2010	Tick	MIT 27	GGII	5
MEL	2010	Tick	MIT 24	GGI_A_	6

*Abbreviations: YB* Yellow Bay, *WHI* Wild Horse Island, *MEL* Melita Island

^a^Pine squirrel (*Tamiasciurus hudsonicus*)

^b^ *Ornithodoros hermsi*

^c^Deer mouse (*Peromyscus maniculatus*), PCR on sample, no isolate

GGI_A_ spirochetes were found on both Wild Horse and Melita Islands but not the mainland, while GGI_B_ spirochetes were found only at Yellow Bay (Table 3). GGII spirochetes were the most abundant group encountered (71 % of isolates) and were found on both islands and Yellow Bay. GGII spirochetes (WHS-40, WHS-81, WHS-88) isolated from three pine squirrels on Wild Horse Island were identical to spirochetes (LAK-3 and LAK-5) isolated from two patients infected there. Additionally, GGI_A_ spirochetes (WHS-90 and WHT-8) isolated from a pine squirrel and *O. hermsi* tick from Wild Horse Island were identical to isolates (LAK-4 and LAK-6) from two other patients infected on this island.

Three pine squirrels were co-infected at the time of capture with spirochetes belonging to different genomic groups. A pine squirrel on Melita Island was infected with GGI_A_ and GGII spirochetes while two squirrels at Yellow Bay were infected with GGI_B_ and GGII spirochetes. The co-infections were identified by comparing DNA sequences of the spirochetes isolated in culture to sequences obtained directly from pine squirrel blood from which the isolates originated.

### Polymorphisms in the variable tick protein (Vtp)

**Fig. 6:**
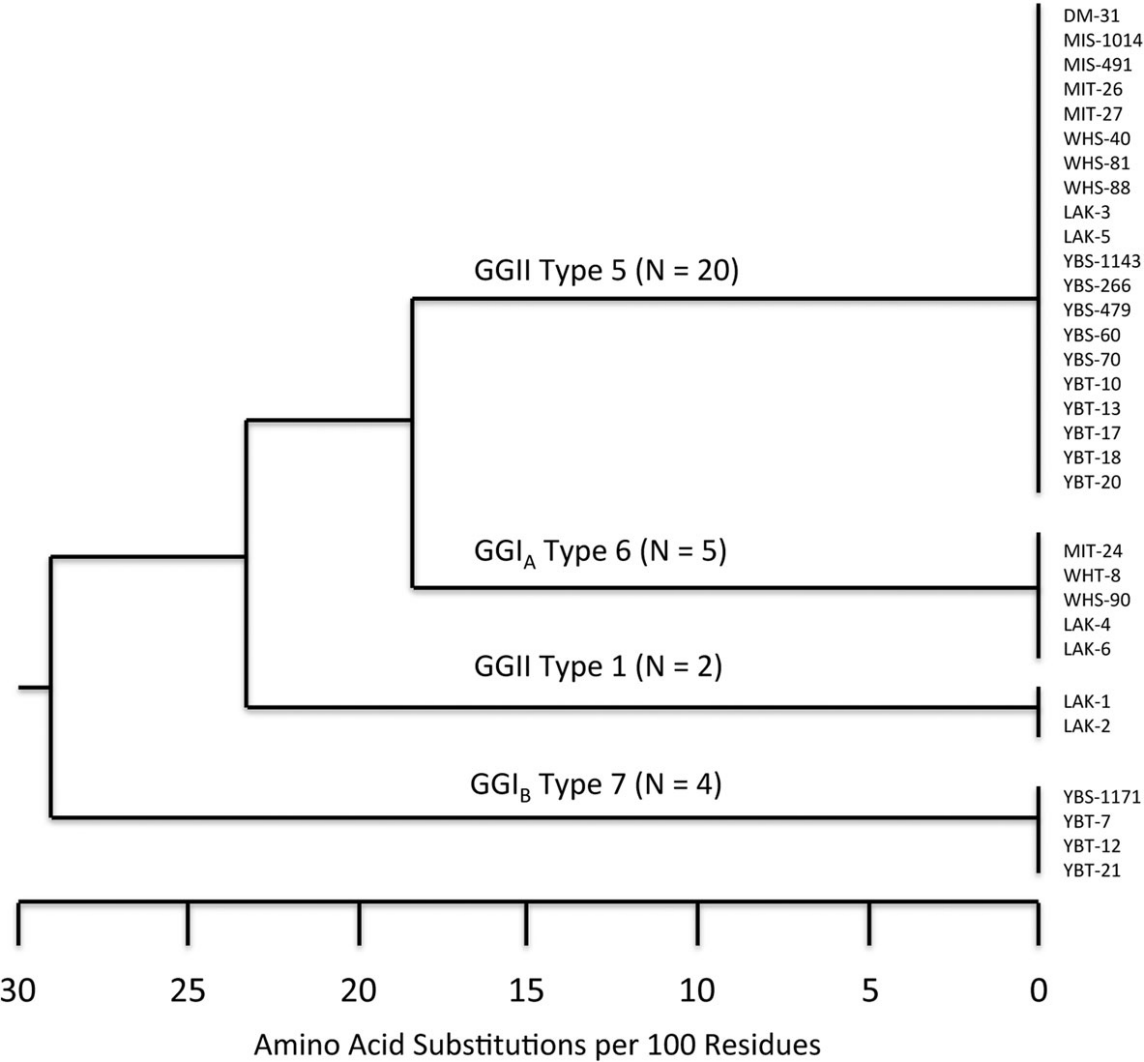
Diversity of the variable tick protein (Vtp) for *Borrelia hermsii* from Flathead Lake, Montana. The tree was constructed with Vtp amino acid sequences (minus the identical signal peptide) for spirochetes infecting pine squirrels (*Tamiasciurus hudsonicus*), ticks (*Ornithodoros hermsi*), one deer mouse (*Peromyscus maniculatus*), and isolates from human patients from previous studies. Sequences were aligned using CLUSTAL W and the tree was constructed using the neighbor-joining algorithm [17]

DNA sequences of the gene that encodes the variable tick protein (Vtp) identified four *vtp* alleles among the 31 samples. The complete open reading frames contained 627, 636, 642, or 648 bp, and the predicted amino acid sequences segregated the proteins into four antigenic groups. These groups included Type 1, 5, 6 and 7 described previously for *B. hermsii* isolated from other locations [15] (Fig. 6). All 31 Vtp sequences contained the identical signal peptide including the first cysteine residue: MKKNTLSAILMTLFLFISC. Within each antigenic group, the DNA and deduced amino acid sequences were identical. Between the groups, the amino acid sequences [minus the identical signal peptide] shared 58.5 to 71.7 % identity. Spirochetes producing different Vtp types were found at each site where spirochetes were collected, with three Vtp types (Type 1, 5, 6) found on Wild Horse Island (Table 3). Spirochetes producing Vtp Type 5 were the most prevalent (20 of 31 samples; 65 %) and were found on both islands and the mainland (Table 3). DNA sequences of the *vtp* gene have been deposited in GenBank with the following accession numbers: KX171844–KX171872.

### Experimental infections of deer mice

**Fig. 7:**
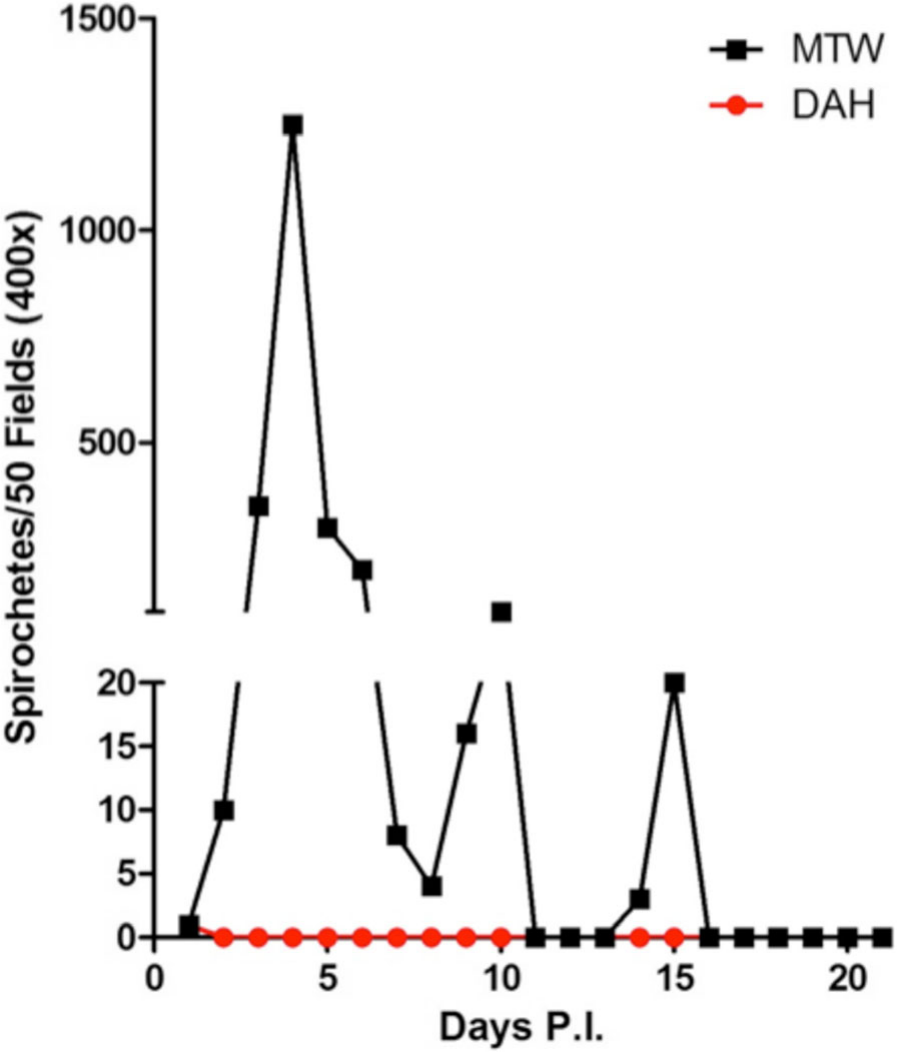
Susceptibility of deer mice (*Peromyscus maniculatus*) to infection with GGI_A_ and GGII *Borrelia hermsii* by needle inoculation. Single mice inoculated with *B. hermsii* DAH (GGI_A_) (*red* circle) or *B. hermsii* MTW (GGII) spirochetes (*black* box). Quantification of the spirochetes is described in the methods. *Abbreviation*: P.I., post-inoculation

Our finding of the deer mouse on Wild Horse Island infected with *B. hermsii* belonging to GGII led us to test the susceptibility of this species to spirochetes in the two genomic groups. In the pilot experiment with single deer mice infected by needle inoculation, only one spirochete was observed in the blood 1 day after infection in the mouse inoculated with *B. hermsii* DAH (GGI_A_), and no spirochetes were observed again during the remaining 20 days (Fig. 7). In contrast, the deer mouse inoculated with *B. hermsii* MTW (GGII) became spirochetemic the next day and had spirochetes detectable in its blood for 11 of the next 15 days. Following the initial peak in spirochetemia on day four, two relapses were observed with subsequent peaks in spirochetemia on days 10 and 15.

**Fig. 8:**
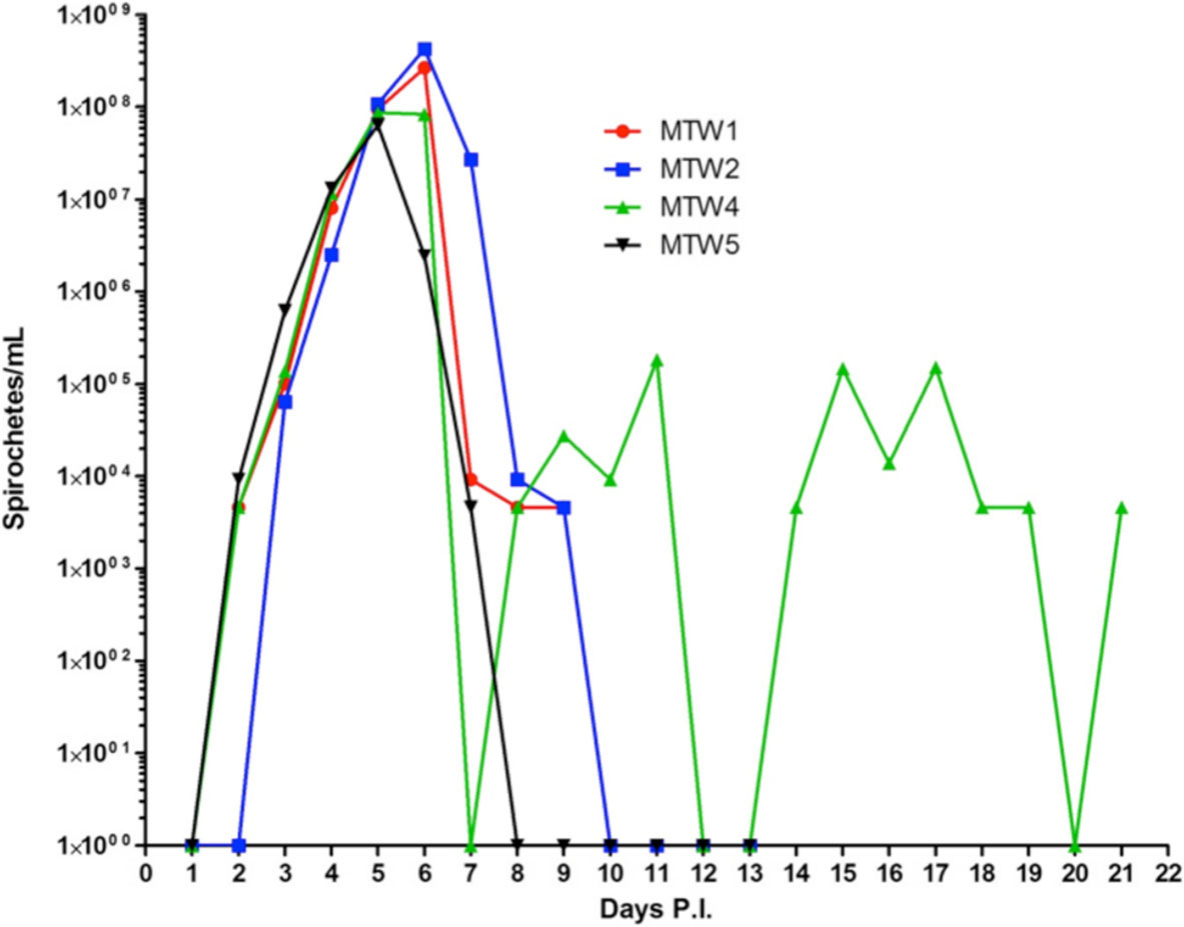
Susceptibility of deer mice (*Peromyscus maniculatus*) to infection with GGI_A_ and GGII *Borrelia hermsii* when fed upon by infected ticks. Only the four mice fed upon by *Ornithodoros hermsi* infected with *B. hermsii* MTW (GGII) became spirochetemic. Three of these mice died on day 11 leaving only one mouse for the remainder of the experiment. No spirochetes were observed in any of the five mice fed upon by ticks infected with *B. hermsii* DAH (GGI_A_) during the 21 days following tick bite and these results are not shown. Quantification of the spirochetes is described in the methods. *Abbreviation*: P.I., post-inoculation

Given the striking difference in infectivity of the two strains of *B. hermsii* shown in the first experiment, we tested more deer mice by exposing them to infected *O. hermsi*. None of the five deer mice fed upon by ticks infected with *B. hermsii* DAH (GGI_A_) had detectable spirochetemias during the 21 days of observation, despite having been fed upon by 51 to 88 ticks each. One white mouse fed upon by another cohort of ticks infected with DAH became spirochetemic, which demonstrated that these ticks were infected. The four deer mice that survived being fed upon by ticks infected with MTW (GGII) spirochetes reached peak spirochetemias five or six days later (Fig. 8). Spirochete cell densities in these mice became extremely high, reaching > 10^8^ spirochetes/ml. Three animals expired on day 11 during anesthesia and blood collection. The deer mouse that survived until day 21 underwent two complete relapses and spirochete density was increasing again on the final day of the experiment. Together, these experiments demonstrated a striking difference in the susceptibility of deer mice to infection with *B. hermsii* depending on the strain and genomic group of the spirochete.

## Discussion

This investigation was prompted by eight people contracting tick-borne relapsing fever in 2002 and 2004 while occupying two cabins on Wild Horse Island in Flathead Lake, Montana [8, 13, 14]. *Borrelia hermsii* was isolated from the patients’ blood and *O. hermsi* ticks were found in one of the cabins [8, 13]. These findings identified a new endemic focus of tick-borne relapsing fever that warranted further investigation.

One primary objective was to identify the small mammals that were hosts for spirochetes and ticks. For this, we live-trapped small mammals, collected blood samples, used microscopy for the direct detection of spirochetes, and performed serological tests to expand our surveillance for the species and number of individuals involved. While only 2.9 % of the animals were infected when captured, nearly 10-times more individuals (27.5 %) were seropositive. Also, only two species had individuals infected when captured yet nine species had seropositive animals. Infected animals were found at three sites while seropositive animals were found at all five sites. Thus our serological tests provided a more comprehensive picture of the number and diversity of small mammal species involved in the circulation of *B. hermsii* at Flathead Lake. These results are similar to what we found when investigating tick-borne relapsing fever spirochetes in small mammals in Mali, West Africa [26]. Compared to the examination of blood smears for current infection, serological tests showed that a greater number of species, individuals, and villages were involved in the circulation of spirochetes, in this case *Borrelia crocidurae*.

Wild Horse and Melita Islands are isolated from the surrounding mainland and have a greatly reduced diversity of small mammal species that include just one or two species of rodents, pine squirrels and deer mice. Chipmunks, which play a primary role as vertebrate hosts for *B. hermsii* elsewhere [10, 27–31], are absent on the islands. Additionally, Douglas or Tamarack squirrels (*Tamiasciurus douglasii*), which are also natural hosts for *B. hermsii* in California [32], do not occur in Montana. Since pine squirrels had never been proven to be natural hosts for the spirochete, we investigated the role this species played in perpetuating the spirochetes on the islands versus mainland sites where host diversity was much greater.

One striking result from our study was the high degree of involvement that pine squirrels had as vertebrate hosts for *B. hermsii*, and through this spirochete’s specific tick vector, hosts for *O. hermsi* as well. On the two islands, most pine squirrels we captured (*n* = 81) were either spirochetemic or seropositive. Several animals were concurrently infected and seropositive, which could have been due to our sampling these animals long enough into their first infection that the pine squirrels had seroconverted but not yet cleared the infection. Alternatively, these individuals may have been seropositive from a previous infection, and infected a second time with a strain to which they were not immune (discussed later).

In previous studies, pine squirrels were found at sites during environmental assessments performed after outbreaks of tick-borne relapsing fever caused by *B. hermsii*, but these animals were not sampled for infection [33–36]. Because pine squirrels were present at a Boy Scout camp in eastern Washington during a large outbreak of tick-borne relapsing fever in 1968 [33], Burgdorfer & Mavros [37] included them with seven other rodent species to test their susceptibility to infection with *B. hermsii* in the laboratory. The three pine squirrels tested, which were infected by injection or tick feeding, yielded the highest and longest lasting spirochetemias compared to the other species examined. However, the squirrels died 17 to 37 days after being infected, which may have been due to the spirochete infections or complicated by these animals’ excitableness in captivity [37]. Yet, their prolonged and high spirochetemias suggested that pine squirrels were competent hosts for infecting ticks that feed on them. Such prolonged spirochetemias might also explain our finding of pine squirrels that were simultaneously infected and seropositive.

The death of the pine squirrels after initiation of their infection with *B. hermsii* in the laboratory [37] runs counter to our observations of the high prevalence of seropositive animals in the field, that is, animals that survived their infection. One explanation might involve differences in the virulence of spirochetes belonging to different groups when infecting different host species, or the relative abundance of the different genomic types of *B. hermsii* present at our study sites. Burgdorfer & Mavros infected animals with *B. hermsii* that originated from infected ticks collected during the investigation of the outbreak in eastern Washington [33, 37]. Stoenner established this spirochete in culture [38], and later designated the strain HS1 [39], which we now know is a GGI_A_ spirochete [8, 15]. Of the 12 isolates of *B. hermsii* we characterized from infected pine squirrels, ten (83 %) were GGII spirochetes, while one isolate represented GG1_A_, to which HS1 also belongs, and one GG1_B_. The preponderance of GGII spirochetes isolated from pine squirrels may simply reflect their greater abundance than GGI spirochetes at our study sites, as 61 % (11 of 18) of spirochetes we isolated from ticks and patients were also GGII spirochetes. Yet, the notion that GGI spirochetes may be more virulent in pine squirrels than are GGII spirochetes, and thus less likely to be encountered in infected animals due to a higher mortality rate, is worthy of future investigations in the laboratory.

Deer mice were the most abundant species we captured at the sites where they occurred, but we encountered only one spirochetemic animal in the three seasons of field work and overall, only 5.5 % of these mice were seropositive. Yet, even the one infected deer mouse ran counter to the results of Burgdorfer & Mavros, who concluded that deer mice were not susceptible to infection with *B. hermsii* [37]. As we discussed above, the strain of *B. hermsii* that Burgdorfer & Mavros used was HS1, a GGI_A_ spirochete. The deer mouse on Wild Horse Island was infected with GGII spirochetes, which led us to test the susceptibility of these mice in the laboratory to infection with *B. hermsii* representing the two genomic groups of spirochetes. Although the number of animals we used was small, the results of the needle inoculation and tick feeding experiments were unambiguous. The deer mice we challenged were refractory to infection with GGI_A_ *B. hermsii*, which confirmed the results of Burgdorfer & Mavros 45 years ago [37], but these animals were highly susceptible to infection with GGII spirochetes. A recent model predicted that the addition of nonsusceptible deer mice to a susceptible pine squirrel population would reduce *R*_*0*_ for *B. hermsii* GGI_A_ spirochetes circulating in the environment [40]. Our study complicates such a model by showing the presence of multiple genome groups of spirochetes in a single focus.

Our serological results show that only a small percentage of deer mice had been infected prior to their capture. The susceptibility experiments suggest these animals had been infected with GGII spirochetes however, our serological tests do not discriminate between the genomic groups of *B. hermsii* that caused the infection. In the only other large serological study investigating the presence of anti-*B. hermsii* antibodies in wild rodents, only 2 of 133 (1.5 %) deer mice tested in California were seropositive [29]. Yet, during a site investigation on Mt. Wilson in Southern California where *O. hermsi* ticks were infected with GGII spirochetes (*B. hermsii* MTW), two of only five brush mice (40 %) (*Peromyscus boylii*) tested were seropositive and broadly reactive by immunoblot analysis [9]. The white-footed mouse (*Peromyscus leucopus*), which is an important vertebrate reservoir for the Lyme disease spirochete *Borrelia burgdorferi* [41], is susceptible to experimental infection with *B. hermsii* GGI spirochetes [42, 43], in contrast to deer mice (*P. maniculatus*). Although the geographical distribution of white-footed mice lies to the east and outside the range where *B. hermsii* is endemic [8, 44], more studies with this and other species of *Peromyscus* are needed to elucidate the mechanisms that control the difference in host species susceptibility to infection with these closely related but genetically distinct relapsing fever spirochetes.

Clearly, the abundance and enzootic involvement of pine squirrels made them the best sentinel species to detect the presence of *B. hermsii* circulating in the habitats we sampled, and on Melita Island, they comprised essentially the only species. On Wild Horse Island where only pine squirrels and deer mice occurred (excluding the single vole captured), a pine squirrel was approximately 22 times more likely to be seropositive than was a deer mouse (95.1 % versus 4.4 %, respectively). Several factors may contribute to this difference. First, pine squirrels are susceptible to infections with both genomic groups of *B. hermsii*, while deer mice are refractory to GGI_A_ spirochetes (this study). Thus pine squirrels have a greater potential to seroconvert when infected with *B. hermsii* regardless of the genomic group of spirochetes to which they are exposed. Second, pine squirrels live longer than deer mice, and have a greater potential to have seropositive individuals accumulate in the population from 1 year to the next. On Cedar Island, another small (9.6 ha) uninhabited island in Flathead Lake northeast of Wild Horse Island, pine squirrels were studied for 13 consecutive years [45]. In spite of their potential longevity of 9 to 10 years in captivity [46], 67 % of the pine squirrel population on Cedar Island lived less than 12 months, although many animals surviving through the next summer lived up to 7 years [45]. In contrast, *Peromyscus* species rarely live longer than 1 year in the wild [47], and natural populations usually have nearly a complete annual turnover [44]. Third, pine squirrels are diurnal [46] and usually remain in their nest at night when the nocturnal *O. hermsi* ticks feed, while deer mice are nocturnal [48], leaving their nests at night when ticks become active. The broader susceptibility to spirochete infection, greater longevity and diurnal behavior of pine squirrels may contribute to their greater involvement as hosts for *B. hermsii* compared to deer mice, and also make them ideal targets for serosurveillance investigations.

Pine squirrels were also heavily involved as hosts for the spirochetes on the mainland, as were three additional sciurid species not present on the islands: the red-tailed chipmunk, yellow-pine chipmunk, and northern flying squirrel. The red-tailed chipmunk was the most abundant species of chipmunk we captured, and nearly half (47.7 %) of the individuals we sampled were seropositive. Yellow-pine chipmunks were far less abundant and rarely captured, yet two of only five individuals we tested were seropositive. This species extends south into the northeastern mountainous half of California [49], where two serological surveys found 36 % (4 of 11) and 47.2 % (34 of 72) of these animals seropositive with anti-*B. hermsii* antibodies [28, 29]. Subsequent to our field study at Flathead Lake, we found seropositive yellow-pine and red-tailed chipmunks in the Bitterroot Valley to the south in western Montana, and one yellow-pine chipmunk was infected with a GGI_A_ *B. hermsii* [31]. Burgdorfer & Mavros showed that yellow-pine chipmunks are susceptible to infection with *B. hermsii* [37], again using the GGI_A_ spirochete (HS1) discussed above. Interestingly, all *B. hermsii* found in yellow-pine or other species of chipmunks during other field studies, when genetically typed, have been GGI spirochetes [8, 10, 30, 31, 50].

The high prevalence of seropositive northern flying squirrels (90.9 %; 10 of 11) was also surprising as Burgdorfer & Mavros [37] found this species was not susceptible to infection with *B. hermsii*. These animals are strictly nocturnal but most active before dawn and after dusk, and spend much of the night in their arboreal nests [51], making them susceptible to bites of infected ticks. Just as chipmunks may be susceptible to only GGI spirochetes, northern flying squirrels may only be susceptible to infection with GGII *B. hermsii*. Again, more experimental work in the laboratory is needed to determine the infectivity of the different spirochetes in these additional and potentially important host species.

The bacterial isolates and infected blood samples obtained during this study and our previous human case investigations [8, 13] represent the largest collection of relapsing fever spirochetes ever assembled from a single endemic focus. Yet, we do not presume that our knowledge of the genetic diversity of these spirochetes at Flathead Lake, or elsewhere, is complete. DNA sequence analysis of the spirochetes identified both genomic groups of *B. hermsii* that have been characterized from other locations that span much of the spirochete’s known distribution [8, 9, 15]. However, during our investigation a novel subgroup of GGI spirochetes emerged that we first recognized with spirochetes infecting a pine squirrel at Yellow Bay during our 2008 field season [25]. Based on MLST of the *16S rRNA, flaB, gyrB and glpQ* loci, the pine squirrel was infected with *B. hermsii* that was unique from nearly all other isolates but identical to a spirochete found in the liver of a dead northern spotted owl (*Strix occidentalis*) collected in Washington State 24 years earlier [25, 52]. Subsequently we found another pine squirrel and three ticks infected with the same strain of spirochete, all at Yellow Bay, and herein we designated this novel group as GGI_B_ to distinguish these spirochetes from the other members of GGI, now designated GGI_A_. Spirochetes in this newly recognized group were also found previously in six *O. hermsi* ticks collected at Eagle Lake, Lassen County, California, in 1995 [53]. PCR and DNA sequence analysis of 211 bp of an internal region of the *B. hermsii flaB* gene, which was one of our gene targets for MLST, identified the 9-bp deletion not present in either GGI_A_ or GGII spirochetes [25, 53]. The complete chromosomal sequence for one of these GGI_B_ isolates from a Yellow Bay tick (YBT-12) was determined and is available in GenBank (CP005706).

GGI_A_ and GGII spirochetes were each isolated from ticks, pine squirrels and humans on Wild Horse Island (this study and [8, 15]), and among the GGII isolates from humans, two genotypes were present. Isolates LAK-1 and LAK-2 from two human patients were unique from the other isolates found on the island but identical to isolates from a domestic dog (WCB-1) infected in central Washington State [54] and a human patient (SIL) infected in the northern-most county of Idaho [15]. Two genomic groups of the spirochete were also isolated from Melita Island and on the mainland at Yellow Bay. Thus each of the three sites where spirochetes were found had the two primary genomic groups of *B. hermsii* present.

Two pine squirrels at Yellow Bay were co-infected with GGI_B_ and GGII spirochetes and one pine squirrel on Melita Island was co-infected with GGI_A_ and GGII spirochetes. Our previous finding of different genomic groups of *B. hermsii* isolated from patients having slept together in the same bed on Wild Horse Island [8] prompted additional studies to examine the ability of single *O. hermsi* ticks to become co-infected with two strains of *B. hermsii* belonging to GGI_A_ and GGII spirochetes. When *O. hermsi* larvae were infected by immersion in liquid cultures containing spirochetes belonging to both genomic groups, these ticks became co-infected and subsequently transmitted both types of spirochetes when fed as nymphs on mice [55]. Nymphal ticks also became co-infected when fed sequentially on mice infected with the different genomic groups of *B. hermsii*, and were later able to transmit one or both types of spirochetes during single feedings on mice [56]. None of the ticks from Flathead Lake that we examined was co-infected, but recently one such tick was found during an environmental assessment associated with a human case of relapsing fever in the Bitterroot Valley, Montana [31]; one of nine ticks collected transmitted GGI_A_ and GGII spirochetes when fed on a single mouse in the laboratory. Thus our finding of pine squirrels co-infected with different strains of *B. hermsii* at the time of their capture could have resulted from these animals either being fed upon by multiple ticks infected with different strains, or becoming infected by a single, co-infected tick.

The large collection of novel isolates of *B. hermsii* obtained from our study sites provided the opportunity to examine the diversity of Vtp types produced by these spirochetes. This surface protein is produced by *B. hermsii* while residing in the tick’s salivary glands [57], is essential for spirochete infectivity in mammals when transmitted by tick-bite [22], is polymorphic [15], and produces a protective antibody response in experimentally vaccinated mice challenged by tick bite [20]. On Wild Horse Island, the 12 isolates of *B. hermsii* comprised three Vtp types (Table 3) that shared 65 to 72 % amino acid identity. If Vtps representing each of these types were used as a vaccine, they would prevent infection with spirochetes producing the same Vtp but not to spirochetes producing the other antigenically different types [20]. We do not know the magnitude of the specific anti-Vtp antibody response produced in pine squirrels, or any mammalian hosts, following a single natural infection with *B. hermsii*. Vtp is switched off by spirochetes in mammals soon after transmission by tick bite, and is replaced by one of the many bloodstream variable major proteins (Vmps) [22, 57]. Whether or not a single infection is sufficient to produce a protective anti-Vtp antibody response in pine squirrels or other mammals to prevent reinfection with a strain of *B. hermsii* producing the same Vtp is not known. Such questions need to be addressed experimentally in the laboratory. However, given the immunogenicity of Vtp [20, 58] and borreliacidal activity of anti-Vtp antibodies [20], immune pressure of the vertebrate host population may be one driving force for the selection for, and maintenance of, multiple sympatric antigenic types of Vtp [58], as we found in this study. The balanced polymorphism in the orthologous protein OspC produced by the Lyme disease spirochetes has been the topic of many investigations [59–63].

During the Last Glacial Maximum approximately 15,000 years ago, our study sites were under the Flathead Lobe, one of the southern-most extensions of the Cordilleran Ice Sheet [64]. With the retreat of the ice to the north and the ecological succession that followed, pine squirrels and other small mammals, ticks, and spirochetes radiated from the south to colonize these new habitats. Spirochetes representing the three basic genomic divisions of *B. hermsii* found to the south are present in the Flathead Lake foci, and we believe our DNA sequence data show that spirochetes were introduced onto Wild Horse Island at least three times in the past, and include representatives of GGI_A_ and two genotypes of GGII.

Introductions of *B. hermsii* to Wild Horse and Melita Islands would have required the dispersal of infected vertebrates or ticks from the mainland. Terrestrial mammals could have swum from the mainland or crossed a temporary land bridge of ice when the lake froze during the winter [65]. In more recent times, humans may have inadvertently introduced infected rodents and ticks during their long history of activity to and from the islands [65]. Flying vertebrates, which we did not include in our work at Flathead Lake, might also have introduced spirochetes and ticks to the islands. *Ornithodoros hermsi* has been found in the nests of a variety of wild birds [13, 66–68], and these ticks will feed on chickens (*Gallus gallus*) and bobwhite quail (*Colinus virginianus*) in the laboratory [8]. The northern spotted owl found dead in Washington and infected with *B. hermsii* [25, 52], further supports the notion that birds may contribute to the spread of this spirochete. Finally, bats may also disperse *O. hermsi* in nature. Longanecker [67] found several *O. hermsi* in tree snags and attics occupied by bats, with *O. hermsi* attached to one bat. While these ticks are fast-feeders and normally do not remain attached to their hosts for more than 60–90 min, ticks that feed at night on nocturnal birds such as owls, and bats, may be subjected to short distance, incremental movements over the land. Additional studies are needed to investigate the potential role of aerial vertebrates as potential hosts of *B. hermsii*.

We wondered if the striking difference in diversity of small mammal species on the islands (low species diversity) compared to the mainland (high species diversity) might provide insights into the popular current hypothesis of the dilution effect, which states that increased diversity of host species reduces the risk of humans to infection with certain zoonotic pathogens [69, 70]. The concept originated during early attempts to control human malaria by placing cattle near people as alternate hosts for the mosquito vectors to feed upon. This dilution of humans as hosts by the addition of cattle reduced the biting rate of plasmodium-infected mosquitos on people, and this method of control was named zooprophylaxis [71]. Much recent interest in the dilution effect has been directed at Lyme disease, in which the risk for human infection increases with a higher prevalence of nymphal *Ixodes scapularis* ticks infected with *B. burgdorferi* [72, 73]. The dilution effect hypothesis predicts that a community with only one or a few highly spirochete-competent vertebrate host species would produce a greater prevalence of *B. burgdorferi*-infected ticks than would a more diverse composition of hosts, in which some species are not susceptible to spirochete infection but yet are suitable hosts for the ticks. Such an impact of increased host diversity on the reduced prevalence of nymphal ticks infected with *B. burgdorferi* has been observed in some studies but not others, and the hypothesis in general has been questioned [69, 71–75].

If more *O. hermsi* ticks were infected with *B. hermsii*, the risk of infection to humans living in an endemic area would theoretically increase. While the prevalences of infected ticks we sampled on the islands versus the mainland were 50 and 38 %, respectively, the numbers of ticks were small, the difference in infection was not significant, and the samples originated from few nests. Additionally, even if more *O. hermsi* ticks were infected, the actual risk to humans would unlikely increase at all, given the striking differences in how ixodid and argasid ticks feed [76]. More infected *I. scapularis* nymphs questing for hosts on the open landscape where people are active during the day would increase the risk of infection, while a greater prevalence of infected *O. hermsii* living in pine squirrel nests or tree cavities would unlikely have any impact on the risk of human infection. However, our tick infection data are not adequate to test for the dilution effect caused by the greater diversity of small mammals on the mainland versus the islands.

## Conclusions

We observed a lower seroprevalence among animals at sites with an increase in species diversity, suggesting that a dilution of transmission may occur in highly diverse host communities in which more species are available as hosts for ticks. The prevalence of infected and seropositive pine squirrels were both greater for these animals on the islands versus the mainland, although the differences were not statistically significant. Our results demonstrate that pine squirrels at Flathead Lake are integrally involved in the maintenance cycle of *B. hermsii* and its tick vector *O. hermsi*. On the mainland, other species of sciurids including chipmunks and flying squirrels are also important hosts when present. Future studies should address the potential role of birds and bats as hosts for *B. hermsii*, and to further investigate the susceptibility of various terrestrial host species to infection with spirochetes belonging to different genomic groups.

## Abbreviations

BSK: Barbour-Stoenner-Kelly
GGI: Genomic group I
GGII: Genomic group II
MLST: Multi-locus sequence typing
PBS: Phosphate-buffered saline
TN: Trap night
Vtp: Variable tick protein
